# An Efficient Approach to Obtain Optimal Load Factors for Structural Design

**DOI:** 10.1155/2014/456826

**Published:** 2014-07-15

**Authors:** Juan Bojórquez, Sonia E. Ruiz

**Affiliations:** Institute of Engineering, Universidad Nacional Autonoma de Mexico, Coyoacan, 04510 Mexico City, DF, Mexico

## Abstract

An efficient optimization approach is described to calibrate load factors used for designing of structures. The load factors are calibrated so that the structural reliability index is as close as possible to a target reliability value. The optimization procedure is applied to find optimal load factors for designing of structures in accordance with the new version of the Mexico City Building Code (RCDF). For this aim, the combination of factors corresponding to dead load plus live load is considered. The optimal combination is based on a parametric numerical analysis of several reinforced concrete elements, which are designed using different load factor values. The Monte Carlo simulation technique is used. The formulation is applied to different failure modes: flexure, shear, torsion, and compression plus bending of short and slender reinforced concrete elements. Finally, the structural reliability corresponding to the optimal load combination proposed here is compared with that corresponding to the load combination recommended by the current Mexico City Building Code.

## 1. Introduction

The structural design guidelines can be based on different reliability formats [1], for example, (a) the semiprobabilistic approach [2], (b) first-order and second moments, FOSM [3–5], and (c) load and resistance factor design (LRFD) [6, 7], based on hazard analysis [8, 9] or based on optimization [10–13]. Most of the design codes in the world, including the Mexico City Building Code 2004 (RCDF from its acronym in Spanish), use the load and resistance factor design (LRFD) approach. Load and resistance factors play a significant role in determining the structural reliability. Excessive safety margins increase the construction costs, while insufficient conservatism increases the risk of structural failure. In the past, the calibration of these partial factors was derived from experience and expert judgment. The recent tendency is to preview the design goals, which can be focused on as an optimization problem where the control variables are the partial safety factors. Calibration procedures have been described since the 70s, for example, in [14] and also in [15–19]. The calibration procedure can be seen as a specific optimization process where the control variables are the partial factors of a design code. The choice of an appropriate method is not usually an easy task. In this paper, an efficient optimization procedure is described to find the optimal load factors that will appear in the new version of the Mexico City Building Code. The reliability index *β* [20] is used to derive the load factor combination that should be used for designing structures under flexure, shear, torsion, and compression plus bending. The load factors are calibrated so that the reliability indexes are as close as possible to a target reliability index. The basic combination of dead and live loads is considered. It is proposed that the load factors obtained here be included in the new version of the Mexico City Building Code (RCDF-04) [21].

The study contains three sections. The first reviews the reliability (associated with different limit states) implicit in the Mexico City Building Code. In the second, the calibration procedure is applied to estimate the optimal combination of load factors corresponding to the RCDF-04, based on a target reliability value. A comparison between the reliability of structural sections designed with the combination of loads proposed here and those specified by RCDF-04 is presented in the third section.

## 2. Reliability Analysis

The load and resistance factor design [22] criterion considers that a structural design is satisfactory if the internal forces acting are smaller than or equal to the design resistance of each structural element, which is represented as

(1) $$\begin{array}{c} S_{d}=\gamma S_{n}\leq \phi R_{n}=R_{d}, \end{array}$$

where *S* and *R* stand for the load and resistance forces and subscript *n* denotes the nominal and subscript *d* the design values; *γ* and *ϕ* are the factors accounting for the uncertainties of load and resistance, respectively. The values of *γ* and *ϕ* specified in RCDF-04 are shown in Tables 1 and 2.

**Figure 1:**
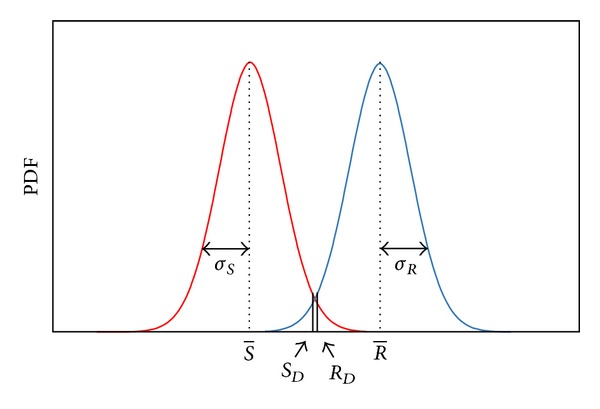
Load-resistance model for structural reliability assessment.

In Figure 1 the load-resistance model for reliability analysis is presented. In this figure the vertical axis represents the probability density function (PDF), and the horizontal axis is the structural resistance (*R*) or the loads (*S*) acting on the structure. $\overset{-}{R}$ and $\overset{-}{S}$ are their mean values, respectively, and *σ*_*R*_ and *σ*_*S*_ their corresponding standard deviations.

### 2.1. Reliability Index *β*

The calibration procedure used here for the selection of optimal partial load factors is based on the structural reliability theory. The reliability index *β* [20], which has proved to be a practical and appropriate link between traditional design procedures and explicit probabilistic design, is used as a measure of the structural reliability. The calibration procedure includes the following steps.

(1) Properties of structural materials and the characteristics of the different cross-sections are simulated by means of a Monte Carlo simulation [23]. The concrete strength *f*_*c*_′, steel yield stress *f*_*y*_, width *b*, height *h*, and cover of the structural sections *r* are considered random variables. Their probability density functions are assumed to be Gaussian [24, 25]. The resistance (*R*) associated with each of the simulated cross-sections is calculated for each limit state (flexure, shear, torsion, and compression plus bending of short and slender columns); then, the mean value $\bar{R}$ and the standard deviation *σ*_*R*_ are estimated.
(2) The design resistance (*R*_*d*_) is calculated (see (1)). It is assumed that *R*_*d*_ is equal to the design load *S*_*d*_. Here, the mean value $\bar{S}$ is taken equal to *S*_*n*_ because it is assumed that the nominal loads have a 50% probability of exceedance corresponding to areas of approximately 36 m^2^ [26], and the coefficient of variation *C*_*s*_ of the loads is obtained as follows [27]:
  (2) $$\begin{array}{c} C_{S}^{2}=C_{\gamma }^{2}+r_{c}^{2}C_{D}^{2}+{\left(1-r_{c}\right)}^{2}C_{L}^{2}, \end{array}$$

where *Cγ*, *C*_*D*_, and *C*_*L*_ are coefficients of variation associated with model uncertainty for dead and live loads, respectively. The following values were assumed in this study: *Cγ* = 0.1, *C*_*D*_ = 0.08, and *C*_*L*_ = 0.18; and *r*_*c*_ is the load ratio given by

(3) $$\begin{array}{c} r_{c}=\frac{DL}{DL+LL}, \end{array}$$

where *DL* represents the dead load and *LL* the live load. The reliability index *β* is defined as [20]

(4) $$\begin{array}{c} \beta =\frac{\overset{-}{R}-\overset{-}{S}}{\sqrt{\left(\sigma_{R}^{2}+\sigma_{S}^{2}\right)}}. \end{array}$$

**Figure 2:**
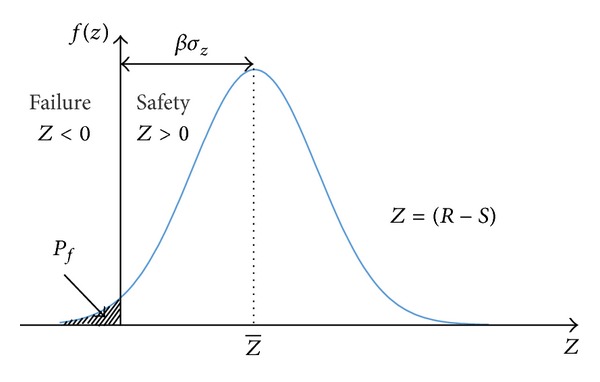
Safety margin distribution.

Figure 2 shows that the *β* value is the distance between the failure region and the mean of the safety margin (*Z*). The index *β* can be used to estimate the probability of failure (*P*_*f*_) [28]:

(5) $$\begin{array}{c} P_{f}=\Phi \left(-\beta \right), \end{array}$$

where Φ(·) is the cumulative distribution function of a Gaussian distribution. The value of *β* indicates the level of structural safety; the higher the value of *β* index is, the lower the probability of failure is.

## 3. Calibration Procedure

Most current design guidelines are largely based on engineering experience and judgment and lead to designs with a generally satisfactory behavior; the structural reliability implicit in those designs is undefined and unknown. The objective of the calibration of codes based on a LRFD format is to provide optimal partial factors for the design of a type of structure, which lead to designs as close as possible to the code objective. The calibration procedure for obtaining the load factors can be seen as an optimization process where the control variables are the factors. In the present study the load factors were calibrated so that the reliability indexes were as close as possible to a target reliability index *β*_*o*_. This can be formulated by means of the following optimization problem [29, 30]:

(6) $$\begin{array}{c} \min W\left(\gamma \right)=\underset{k}{\sum }\underset{j}{\sum }w_{j}{\left(\beta_{k}\left(\gamma \right)-\beta_{o}\right)}_{j}^{2}, \end{array}$$

where *w*_*j*_ are factors indicating the importance of the limit states of interest. For each limit state *j*, *β*_*k*_(*γ*) represents the reliability of the element *k* given the partial safety factor *γ*; *β*_*o*_ is the reliability target index and *W*(*γ*) represents different combinations of load factors. The optimal load factors are obtained by the numerical solution of the minimization problem given by (6).

## 4. Material Characteristics

### 4.1. Concrete Strength

Two types of concrete are considered: ordinary and high-strength. The mean compressive strength $\bar{f_{c}}$ of the ordinary concrete (in the field) is taken as 24.51 MPa, and the standard deviation *σ*_*fc*_ is 3.37 MPa. For the high-strength concrete the values of $\bar{f_{c}}$ and *σ*_*fc*_ are considered to be 59.50 MPa and 5 MPa, respectively [25].

### 4.2. Steel Yield Stress

A bilinear stress-strain relationship is assumed, and Young's modulus is equal to 195,811 MPa. The mean value $\bar{f_{y}}$ is considered equal to 458.8 MPa, the coefficient of variation *C*_*fy*_ = 0.096, and the nominal value *f*_*y*_ = 413.70 MPa [24, 31].

### 4.3. Cross-Section Characteristics

A set of eighteen reinforced concrete elements designed for live plus dead loads was analyzed. Each element was designed with the RCDF-04. The limit states under consideration were flexure, shear, torsion, and compression plus bending. The mean and standard deviation for each section analyzed are shown in Table 3 [32, 33].  Table 4 shows the transverse reinforcement adopted.

## 5. Reliability Indexes 

In this section the compatibility and consistency between the *β* values associated with different limit states are reviewed. The reliability evaluation was carried out for the load ratios *r*_*c*_ (see (3)) commonly used in practice [6]. The intervals of values are from 0.30 to 0.70 for flexure, shear, and torsion and from 0.40 to 0.90 for flexure plus bending. The analysis was performed for a set of eighteen cross-sections, and then the mean value of the *β* index was calculated. The geometric characteristics of sections and material properties were obtained from typical Mexican constructions. The influence of some parameters on the reliability of the elements is discussed in the next sections. It is noticed that the designs were performed using the factors *γ* and *ϕ*, listed in Tables 1 and 2, respectively.

### 5.1. Flexure

**Figure 3:**
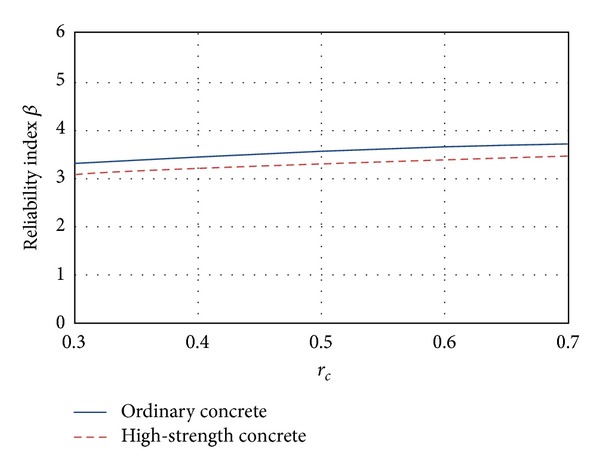
Reliability corresponding to flexure failure mode.

In Figure 3 the mean *β* values corresponding to flexure are presented. The figure shows that the *β* values increase as *r*_*c*_ grows, which means that *β* increases for smaller values of live loads (see (3)). It is noticed that this behavior is undesirable because the uncertainties implicit on live load are higher than those corresponding to dead loads. Also, it can be noticed in Figure 3 that the reliability associated with high-strength concrete sections is smaller than the reliability associated with ordinary concrete sections.

**Figure 4:**
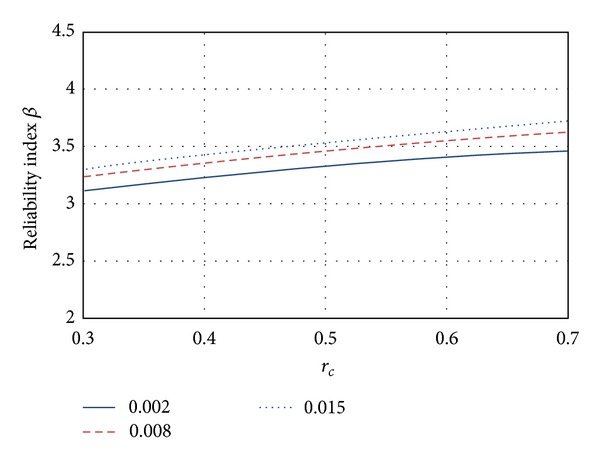
Influence on the structural reliability of the longitudinal reinforcement *ρ*_*g*_.

The influence of the transversal steel reinforcement using ordinary concrete is shown in Figure 4. Three longitudinal steel percentages were used: *ρ*_*g*_ = 0.002, 0.008, and 0.015. It can be seen that when the percentage *ρ*_*g*_ increases, the reliability index *β* becomes higher. The maximum *β* differences for this case are about 10%.

### 5.2. Shear

**Figure 5:**
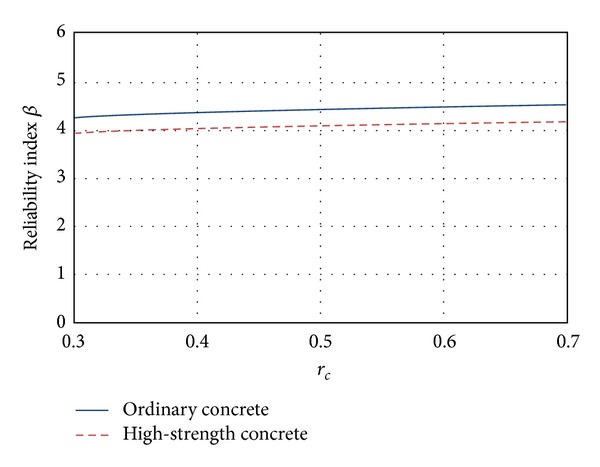
Reliability corresponding to shear failure mode.

Results of the reliability index *β* for shear designs are shown in Figure 5. The reliabilities for these designs are consistent with the corresponding flexure designs because the reliability associated with shear designs is larger than that corresponding to flexure. The increase in the reliability levels for brittle failure modes is achieved by setting a lower resistance factor than that associated with ductile failure modes. It can be seen in Figure 5 that for RCDF-04 the reliability index values *β* increase as the *r*_*c*_ ratio also increases (similar to the case of flexure). As it was described before, this behavior is not desirable because the failure probability tends to increase for higher values of live load. Also it can be observed that the reliability corresponding to high-strength concrete sections is smaller than the one corresponding to ordinary concrete.

**Figure 6:**
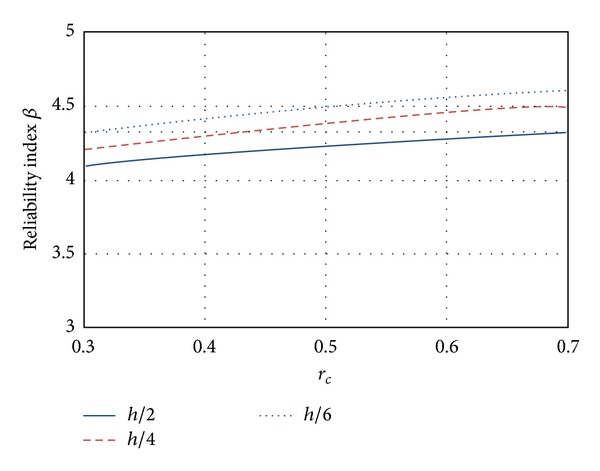
Influence on the structural reliability of the transversal reinforcement.

Figure 6 shows the behavior of the index *β* for elements designed using ordinary concrete and three-different-stirrup spacing, which are indicated as a fraction of the specified *h* value. It can be appreciated that as the spacing of the stirrups decreases, the reliability of the element increases, as expected. The lower reliability curve (indicated by continuous line) corresponds to stirrup spacing equal to *h*/2, while the curve with the greatest values of *β* corresponds to structural elements designed with the minimum spacing (*h*/6).

### 5.3. Torsion

**Figure 7:**
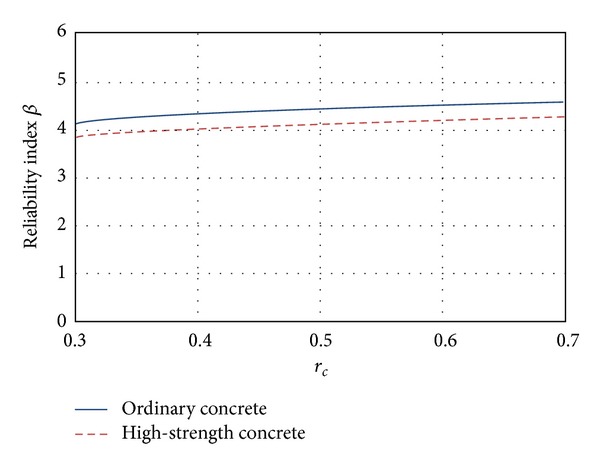
Reliability corresponding to torsion failure mode.

The values of the reliability index *β* for elements designed for resisting torsion forces are congruent with the values obtained for the failure modes previously analyzed (flexure and shear). Ductile failure is associated with higher failure probabilities (flexure), while brittle failure is associated with lower probability of failure (shear and torsion). As observed in Figure 7, the reliability index *β* is smaller for the RCDF-04 as the load ratio (*r*_*c*_) decreases, which is undesirable. Similar to flexure and shear modes, the reliability associated with high-strength concrete is smaller (about 5%) than the reliability associated with ordinary concrete.

**Figure 8:**
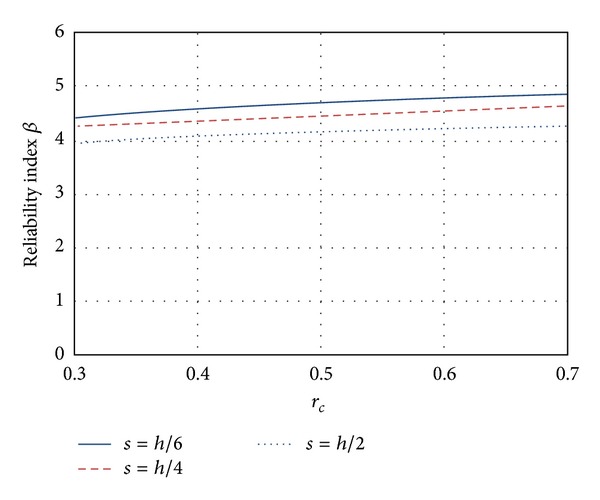
Influence on the structural reliability of the transversal reinforcement.

Figure 8 shows the reliability index *β* for elements designed with ordinary concrete and with stirrup spacing equal to *h*/2, *h*/4, and *h*/6. Reliability increases as the stirrup spacing decreases, as shown in Figure 8. The curve with the greatest reliability index corresponds to a spacing *s* = *h*/6, while the curve with the smallest reliability level corresponds to the maximum value (*s* = *h*/2).

### 5.4. Compression plus Bending

**Figure 9:**
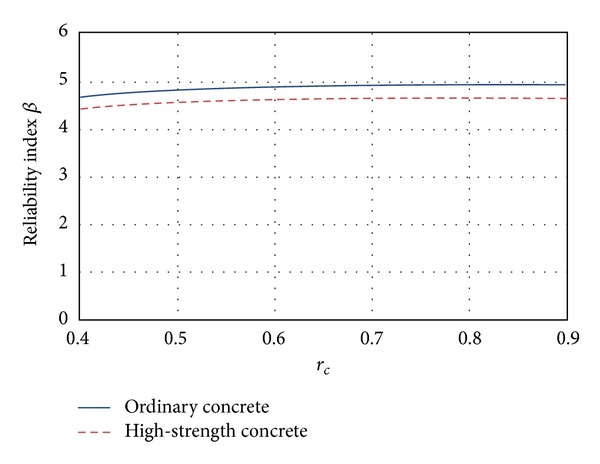
Reliability corresponding to compression plus bending mode (*e* = *e*_*b*_).

The resistance *R* of the element subject to flexure plus bending is obtained as follows:

(7) $$\begin{array}{c} R=\sqrt{P^{2}+{\left(\frac{M}{h_{n}}\right)}^{2}}, \end{array}$$

where *h*_*n*_ is the nominal depth of the section, *P* is the resisting axial load, and *M* is the resisting bending moment associated with an eccentricity *e*. This study considers three eccentricities that correspond to three different zones: zone A corresponds to elements failing in compression (*e* = 0), zone B to elements failing at the balanced condition (*e* = *e*_*b*_), and zone C to those failing under flexure (*e* = *∞*).  Figure 9 illustrates the reliability indexes *β* related to zone B, for a cross-section of 0.4 × 0.75 m. The longitudinal reinforcement is 1.5 percent of the section area, distributed in 4 rod layers. It was observed that the higher the load ratio is, the larger the magnitude of *β* is. Also it can be observed that, for high-strength concrete sections, the structural reliability becomes smaller (about 7%).

**Figure 10:**
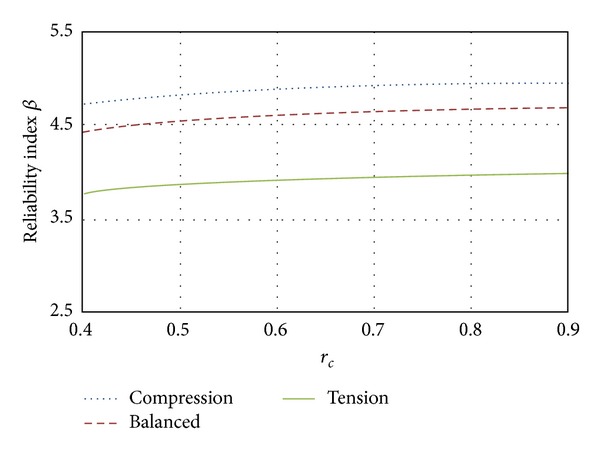
Influence of eccentricity on the structural reliability.

The influence of the eccentricity *e* is analyzed in Figure 10 which corresponds to elements designed with ordinary concrete. In zone A (corresponding to pure compression failure *e* = 0) designs have the highest reliability index *β*. In zone B, corresponding to the balanced condition (*e* = *e*_*b*_), the *β* reliability index is 6% lower than that corresponding to zone A, and for the case in zone C (controlled by pure bending *e* = *∞*) reliabilities present smaller values (80% of that corresponding to zone A).

## 6. Slenderness Ratio

The influence of slenderness ratio on reinforced concrete column reliability has been studied by several authors [34, 35]. In the present study, the effects of slenderness in the strength were considered by means of the following expression, using a numerical integration technique [36]:

(8) $$\begin{array}{c} \Delta_{m}=\frac{l^{2}\left(\phi_{m}+0.25\phi_{e}\right)}{10}, \end{array}$$

in which Δ_*m*_ = lateral deflection at midheight of the column; *ϕ*_*m*_ = curvature at midheight of the column; *ϕ*_*e*_ = curvature at the column ends; *l* = height of the column.

**Figure 11:**
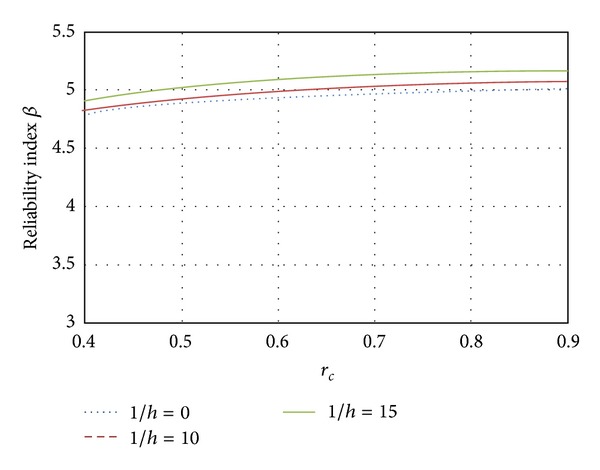
Influence of the slenderness; *e* = 0.01.

**Figure 12:**
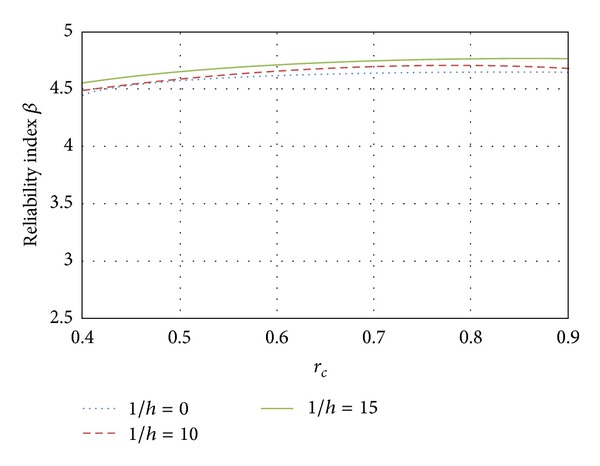
Influence of the slenderness; *e* = *e*_*b*_.

**Figure 13:**
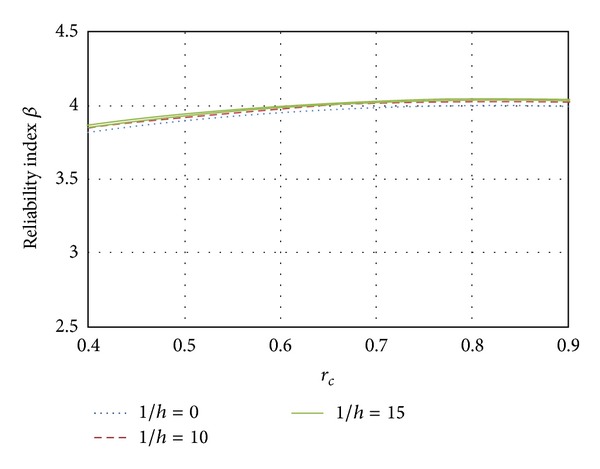
Influence of the slenderness; *e* = *∞*.

Here, the influence of the slenderness ratio on the reliability index *β* was evaluated as a function of the eccentricity. In Figures 11, 12, and 13 results are presented for elements designed with ordinary concrete and slenderness ratios equal to *l*/*h* = 0, *l*/*h* = 10, and *l*/*h* = 15, respectively, where *l*/*h* = 0 represents a short column and *l*/*h* = 15 represents a slender column.

Figure 11 shows the variation for the three slenderness ratios when the element fails in compression. It can also be seen in Figure 11 that as the load ratio increases, the reliability becomes higher. The reliability of slender columns is higher than that corresponding to short columns by about 4% for *l*/*h* = 15 and 2% for *l*/*h* = 10. The results for elements failing close to the balanced condition are shown in Figure 12. Again, it can be observed that the reliability of slender columns is greater than the reliability of short columns; however, this difference is reduced to 2% for columns with *l*/*h* = 15 and less than 1% for columns with slenderness ratio *l*/*h* = 10. When the eccentricity tends to be large (pure bending, see Figure 13), the slender and short columns have similar reliability. The influence of the slenderness ratio decreases as the eccentricity tends to the flexure failure. It is noticed that the difference of *β* between all cases related to slenderness ratios is smaller than 1%.

## 7. Calibration of the Code

In order to obtain the optimal load factors (using (6)), the first step is to calculate the reliability target index *β*_*o*_, which is calculated as the average of the indexes within the interval of *r*_*c*_ values commonly used in practice. The intervals are 0.30 to 0.70 for flexure, shear, and torsion, while for flexure plus bending they are 0.40 to 0.90. The values of (*β*_*o*_)_*j*_ calculated for RCDF-04 are shown in Table 5.

Then, it is necessary to calculate the values of the index *β*_*kj*_ corresponding to different structural elements (*k*) and different limit states (*j*) and assuming different load ratios (*r*_*c*_).

In order to find the optimal values of the load factors, different combinations of dead load factors (*γ*_*D*_) and live load factors (*γ*_*L*_) were assumed in (6). The load factor combinations analyzed were increased from 1.1 to 1.5 for *γ*_*D*_ and from 1.1 to 1.9 for *γ*_*L*_, and the step interval was 0.1. It is noticed that the factors *γ*_*D*_ = 1.4 and *γ*_*L*_ = 1.4, recommended by RCDF-04, are included in this range.

The factors *w*_*j*_ (see (6)) were selected as follows: flexure 0.75, shear 1.0, torsion 1.0, and compression plus bending 0.9. These factors were assumed taking into account that the consequence of a brittle failure (shear or torsion) is more important than that corresponding to ductility failure (flexure and compression plus bending).

**Figure 14:**
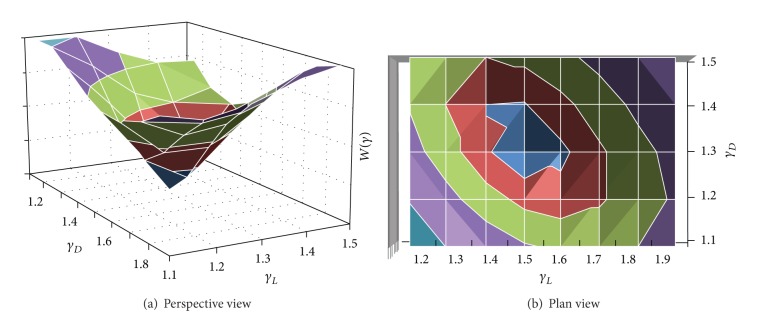
Calculation of optimal load factors.

The results of evaluating (6) for different load combinations are illustrated in Figures 14(a) and 14(b), in which Figure 14(a) corresponds to a perspective view and Figure 14(b) represents the same results seen in plan. The horizontal axes in Figure 14 represent the load combinations considered, and the vertical axis is the result of (6). From Figures 14(a) and 14(b) it can be observed that the minimum value of the summation corresponds to the load combination of *γ*_*D*_ = 1.3 and *γ*_*L*_ = 1.5, which means that this is the optimal combination.

## 8. Reliability Obtained with the Proposed Factors and with Those Specified by RCDF-04

**Figure 15:**
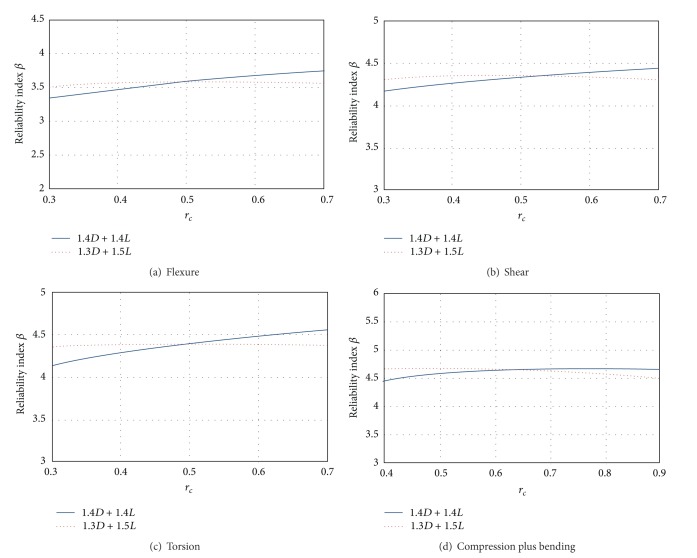
Reliabilities obtained with the *γ* combination recommended by RCDF-04 and with the *γ* combination proposed in this study.

Figures 15(a)–15(d) show a comparison of the proposed load combination (*γ*_*D*_ = 1.3 and *γ*_*L*_ = 1.5, indicated by dotted line) and the *γ* values recommended by RCDF-04 (*γ*_*D*_ = 1.4 and *γ*_*L*_ = 1.4, shown in solid line).  Figure 15(a) corresponds to flexure designs; this figure shows that the load factors combination proposed in this study gives place to an almost uniform reliability index with respect to different load ratios *r*_*c*_. A similar behavior is obtained for the other failure modes. The reliability index *β* obtained using the load factors proposed in this study gives place to similar levels of probability of failure regardless of the load ratio *r*_*c*_, which can be observed in Figures 15(b), 15(c), and 15(d), corresponding to the limit states of shear, torsion, and compression plus bending, respectively.

## 9. Conclusions

1. The values of *β* implicit in structural sections designed for different limit states in accordance with the Mexico City Building Code (RCDF-04) were reviewed. For the cases analyzed, it is concluded that the reliability indexes of the RCDF-04 are consistent for the limit states analyzed, which means brittle failure modes are of more safety than ductile failure modes.
2. It is proposed that the next version of the Mexico City Building Code changes the load factor combination values corresponding to dead load and live load. The proposal is to use *γ*_*D*_ = 1.3 and *γ*_*L*_ = 1.5 instead of *γ*_*D*_ = 1.4 and *γ*_*L*_ = 1.4.
3. The load combination factors recommended in this study have the following advantages.
  - The reliability of structures is nearly uniform for different load ratios when using the proposed combination; however, when using the combination of *γ*_*D*_ = 1.4 and *γ*_*L*_ = 1.4 the structural reliability tends to decrease as the values of load ratios *r*_*c*_ (high live load) decrease, which is undesirable.
  - The factor combination proposed here gives more importance to the variable actions (live load) by means of the factor 1.5 than the factor 1.4 which is now recommended by RCDF-04.

## Figures and Tables

**Table 1:** Load factors, *γ*.

Type of load	RCDF-04
Dead	1.4^∗^
Live	1.4^∗^

^*∗*^ *γ* values should be equal to 1.5 for the design of important constructions.

**Table 2:** Resistance factors, *ϕ*.

Limit state	RCDF-04
Flexure	0.9
Shear	0.8
Torsion	0.8
Compression plus bending	0.8, 0.9

**Table 3:** Characteristics of the elements analyzed.

Dimension	Specified value (m)	Mean value (m)	Standard deviation (m)
Width (*b*)	0.3	0.304	0.0041
Width (*b*)	0.4	0.396	0.0064
Width (*b*)	0.45	0.446	0.0064
Depth (*h*)	0.6	0.596	0.0064
Depth (*h*)	0.75	0.746	0.0064
Depth (*h*)	0.9	0.896	0.0064
Depth (*h*)	1.3	1.298	0.0064
Depth (*h*)	1.6	1.64	0.0062

Cover (*r*)	0.038	0.032	0.011

**Table 4:** Characteristics of transverse reinforcement.

Specified dimension (m)	Transverse reinforcement		
	Stirrup number	Separation of stirrups (s)	Inclination angle (grades)
0.3 × 0.6	2	*h*/2	90
	2.5	*h*/4	90
	3	*h*/6	90

0.3 × 0.75	3	*h*/2	90
	3	*h*/4	90
	3	*h*/6	90

0.3 × 0.9	3	*h*/2	90
	3	*h*/2	60
	3	*h*/2	45

0.4 × 0.9	3	*h*/2	90
	4	*h*/2	90
	5	*h*/2	90

0.45 × 1.3	3	*h*/2	90
	4	*h*/2	60
	5	*h*/2	45

0.4 × 1.6	3	*h*/2	90
	4	*h*/2	90
	5	*h*/2	90

**Table 5:** *β*_*o*_ values for RCDF-04.

Limit state	*β*_*o*_	
	Ordinary	High-strength
Flexure	3.57	3.37
Shear	4.34	4.17
Torsion	4.38	4.21
Flexure plus bending		
Compression	4.87	4.72
Balanced	4.55	4.37
Pure flexure	3.93	3.81
